# Cell fusing agent virus isolated from Aag2 cells does not vertically transmit in *Aedes aegypti* via artificial infection

**DOI:** 10.1186/s13071-023-06033-3

**Published:** 2023-11-06

**Authors:** Ningxin Zhou, Enjiong Huang, Xiaoxia Guo, Yiping Xiong, Jingwen Xie, Tong Cai, Yutong Du, Qixing Wu, Sihan Guo, Wanrong Han, Hengduan Zhang, Dan Xing, Tongyan Zhao, Yuting Jiang

**Affiliations:** 1 Public Health School of Fujian Medical University, Fuzhou, 350122 China; 2 Department of Vector Biology and Control, State Key Laboratory of Pathogen and Biosecurity, Beijing Institute of Microbiology and Epidemiology, Beijing, 100071 China; 3 Fuzhou International Travel Healthcare Center, Fuzhou, 350001 China; 4 Artemisinin Research Center, Guangzhou University of Chinese Medicine, Guangzhou, 510405 China; 5 Life Science College, Southwest Forestry University, Kunming, 650224 China

**Keywords:** Cell fusing agent virus, *Aedes aegypti*, Intrathoracic injection, Vertical transmission

## Abstract

**Background:**

Cell fusing agent virus (CFAV) was the first insect-specific virus to be characterized, and has been reported to negatively influence the growth of arboviruses such as dengue, Zika, and La Cross, making it a promising biocontrol agent for mosquito-borne disease prevention. *Aedes aegypti* Aag2 cells were naturally infected with CFAV. However, the ability of this virus to stably colonize an *Ae. aegypti* population via artificial infection and how it influences the vector competence of this mosquito have yet to be demonstrated.

**Methods:**

CFAV used in this study was harvested from Aag2 cells and its complete genome sequence was obtained by polymerase chain reaction and rapid amplification of complementary DNA ends, followed by Sanger sequencing. Phylogenetic analysis of newly identified CFAV sequences and other sequences retrieved from GenBank was performed. CFAV stock was inoculated into *Ae. aegypti* by intrathoracic injection, the survival of parental mosquitoes was monitored and CFAV copies in the whole bodies, ovaries, and carcasses of the injected F0 generation and in the whole bodies of the F1 generation on different days were examined by reverse transcription-quantitative polymerase chain reaction.

**Results:**

The virus harvested from Aag2 cells comprised a mixture of three CFAV strains. All genome sequences of CFAV derived from Aag2 cells clustered into one clade but were far from those isolated or identified from *Ae. aegypti*. Aag2-derived CFAV efficiently replicated in the mosquito body and did not attenuate the survival of *Ae. aegypti*. However, the viral load in the ovarian tissues was much lower than that in other tissues and the virus could not passage to the offspring by vertical transmission.

**Conclusions:**

The results of this study demonstrate that Aag2-derived CFAV was not vertically transmitted in *Ae. aegypti* and provide valuable information on the colonization of mosquitoes by this virus.

**Graphical abstract:**

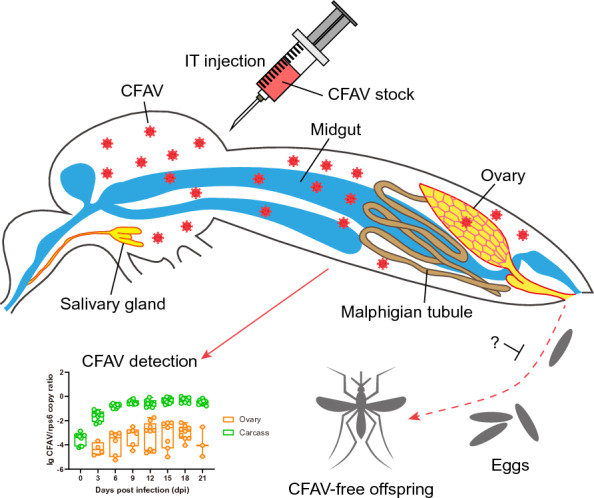

**Supplementary Information:**

The online version contains supplementary material, which is available at 10.1186/s13071-023-06033-3.

## Background

Mosquitoes can transmit various pathogenic viruses, such as dengue virus (DENV), Zika virus (ZIKV) and chikungunya virus, which can cause serious disease in humans [1]. Viruses transmitted between mosquitoes and humans or other vertebrates are termed arthropod-borne viruses (arboviruses). Viruses that only infect mosquitoes or insect cell lines, such as cell fusing agent virus (CFAV) [2], Phasi Charoen-like virus [3] and Nhumirim virus [4], are termed insect-specific viruses (ISVs). An increasing number of studies have shown that ISVs affect the vector competence of mosquitoes for arboviruses in a complicated manner, and that they have potential for use in the prevention of arbovirus transmission [5, 6].

The first ISV, which was isolated from an *Aedes aegypti* cell line in 1975, caused cell fusion when inoculated into *Aedes albopictus* cells, and was subsequently named cell fusing agent virus [2]. Interactions between CFAV and other arboviruses that have been identified in vitro and in vivo can lead to alterations in the replication or dissemination of arboviruses. Zhang et al*.* [7] found that CFAV and DENV enhanced their mutual replication in *Ae. aegypti* Aa20 cells. However, another study [8] reported that CFAV inhibited DENV-1 and ZIKV replication in *Ae. albopictus* C6/36 cells. Schultz et al*.* [9] found that co-infection with CFAV and Phasi Charoen-like virus could inhibit the replication of ZIKV, DENV, and La Cross virus in* Ae. albopictus* Aa23 cells. These contrasting results might be due to the use of different virus strains or cell types. In addition to the in vitro studies cited above, CFAV was also found to inhibit the dissemination of DENV-1 and ZIKV in *Ae. aegypti* mosquitoes [8]. In sum, CFAV was found to compete with arboviruses in most of these studies and showed potential as an agent that may moderate the vector competence of mosquitoes for arboviruses.

Mosquito cell lines are important tools for studying virus-virus interactions or virus-host tropisms, but they lack the sophisticated immune pathways and biological processes present in the mosquitoes from which they are derived, and poorly reflect the interactions that take place in the latter. An in vivo model is thus needed to study the precise effects of CFAV or other ISVs on arboviruses. Baidaliuk et al. [8] investigated how CFAV influenced DENV-1 and ZIKV replication and dissemination in *Ae. aegypti *in vivo. CFAV was used to infect mosquitoes by intrathoracic (IT) injection, then the mosquitoes were fed with an arbovirus blood meal 2 or 6 days after injection [8]. However, it took time for CFAV to disseminate to other tissues (e.g., the salivary gland) to give rise to a stable state of infection. The erratic distribution of CFAV throughout the mosquito body does not reflect the situation in the wild.

*Aedes aegypti* that stably carry CFAV would more accurately reflect the effect of CFAV on the mosquito’s vector competence. There are natural populations of *Ae. aegypti* that carry CFAV and populations that do not in the wild, but differences in their genetic background and the symbiotic microorganisms that they carry can affect experimental results [10, 11]. To eliminate these differences, it is necessary to introduce an exogenous CFAV strain into an *Ae. aegypti* strain that does not carry this virus, to provide a stable CFAV-carrying mosquito colony through offspring screening; wild type mosquitoes with the same genetic background and internal microbial environment be used as the control. The successful introduction of exogenous *Ae. aegypti*-derived CFAV into CFAV-free *Ae. aegypti* via IT was reported by Contraras-Gutierrez et al*.* [12]. However, when we attempted to inoculate Aag2-derived CFAV into *Ae. aegypti* using the same method, we obtained different results. Herein is a detailed description of our experiment.

## Methods

### CFAV

The CFAV used in this study was derived from *Ae. aegypti* Aag2 cells cultured in Schneider’s* Drosophila* Medium (Gibco, Paisley, UK) supplemented with 10% fetal bovine serum (Gibco, Thornton, Australia). The Aag2 cells were disrupted by three cycles of freeze-thawing and the supernatant was harvested as the CFAV stock after centrifugation. The CFAV stock was used for genome sequencing and infection of *Ae. aegypti* Menghai strain. The initial load of CFAV harvested from the Aag2 cells was 5.02 × 10^4^ copies/μL, as determined by reverse transcription-quantitative polymerase chain reaction (RT-qPCR). To obtain a virus stock with a higher load, CFAV was amplified in *Ae. albopictus* C6/36 cells, and the load reached 5.13 × 10^6^ copies/μL. The amplified virus was used to infect *Ae. aegypti* Haikou strain.

### Genome sequencing

CFAV genomic RNA was extracted from the virus stock by using QIAamp Viral RNA Mini Kit (QIAGEN, Hilden, Germany), and then reverse transcribed into complementary DNA (cDNA) using the Eastep RT Master Mix Kit (Promega, Madison, WI). The viral genome was amplified by PCR using 16 pairs of primers (Additional file 1: Table S1) and PrimeSTAR HS DNA Polymerase (TaKaRa, Dalian, China). The amplification products were sequenced by Sangon Biotech (Beijing, China). In the sequencing results, two sites of the 6th PCR segment showed double peaks, indicating the presence of mutations in this region. Then, the 6th PCR products were purified by NuleoSpin Gel and PCR Clean-up Kit (MACHEREY–NAGEL, Düren, Germany) and cloned into a vector using pEASY-T1 Simple Cloning Kit (TransGene Biotech, Beijing, China). The recombinant plasmid was transformed into *Escherichia coli* DH5α competent cells (Accurate Biotechnology, Hunan, China), which were then cultured on LB plates. A total of 40 colonies were inoculated into LB broth and incubated with shaking at 37 °C for 8 h. The plasmids of these colonies were purified using SteadyPure plasmid DNA Extraction Kit (Accurate Biotechnology) and sequenced by Sangon Biotech. Finally, 35 valid sequencing results were obtained.

The ends of the CFAV genome were amplified by rapid amplification of cDNA ends (RACE) to obtain the 3' and 5' ends sequences, as described previously [13]. Briefly, a polyadenylated tail was added to the RNA genome by using Poly (A) Polymerase (TaKaRa). Then, first-strand cDNA synthesis, RACE and In-Fusion Cloning of RACE products were performed using SMARTer RACE 5ʹ/3ʹ Kit (TaKaRa), according to the user manual. The RACE products were sequenced at Sangon Biotech. Genome assembly was performed using SnapGene 2.3.2 (Insightful Science). The primers used for RACE are given in Additional file 1: Table S1.

### Phylogenetic analyses

A total of 26 CFAV genome sequences were used in the analyses, including three newly sequenced strains from this study and 23 strains retrieved from GenBank, of which the Mex_AR269 strain was the shortest (9555 nucleotides). Alignment of these sequences was conducted by using the ClustalW function in MEGA X [14]. The phylogenetic analysis of the nucleotide sequences was constructed by using the neighbor-joining method together with 1000 replication bootstrap using MEGA X.

### Mosquitoes

*Aedes aegypti* Menghai strain was collected from Menghai County, Yunnan Province, China (21°57′48″N, 100°27′34″E), in September 2019. *Aedes aegypti* Haikou strain was collected more than a decade ago in Haikou City, Hainan Province, China [15]. The mosquitoes were reared under standard insectary conditions at 26 ± 1 ℃ and 75 ± 5% relative humidity, with a photoperiod of 14 h light:10 h dark. Adult mosquitoes were provided with 8% sucrose solution. The two *Ae. aegypti* strains were proven to be CFAV-free by using RT-qPCR examination and virus isolation in C6/36 cells.

### Infection of mosquitoes

Seven-day-old female *Ae. aegypti* mosquitoes were anaesthetized with CO_2_, placed on a cold tray, and inoculated with 300 nL of CFAV stock or phosphate buffered saline (PBS) by IT injection using a FemtoJet 4i (Eppendorf, Hamburg, Germany). The injected mosquitoes were then reared under standard insectary conditions as described above. CFAV-infected mosquitoes were used for survival monitoring, CFAV detection, or propagation. The Menghai strain was inoculated with CFAV directly harvested from Aag2 cells while the Haikou strain was infected with CFAV that had been amplified in C6/36 cells.

### Mosquito processing and RNA extraction

To detect CFAV in different tissues, the mosquitoes were dissected with sterile dissecting needles, then the ovary and carcass were collected individually and transferred into 1.5-mL microtubes containing 1 mL of RNAiso Plus (TaKaRa). Total RNA was then extracted according to the manufacturer’s instructions. For CFAV detection in the whole body, mosquitoes were directly used for RNA extraction without dissection.

### RT-qPCR detection

CFAV and the host endogenous *rps6* gene were detected using the GoTaq Probe 1-Step RT-qPCR System (Promega). The following were used in the RT-qPCR reactions: 2 μL of RNA template, 10 μL of GoTaq Probe qPCR Master Mix, 0.4 μL of GoScript RT Mix, 1 μL each of forward primer, reverse primer, and probe for CFAV, 1 μL each of forward primer, reverse primer, and probe for *rps6* and 1.6 μL of nuclease-free water to yield a 20-μL final reaction volume. Amplification reactions were performed in the QuantStudio 7 Flex Real-Time PCR system (Thermo Fisher Scientific, Waltham, MA) and programmed as follows: 1 cycle at 45 °C for 15 min, 95 °C for 10 min, 40 cycles at 95 °C for 15 s and 60 °C for 1 min. Virus RNA copies were calculated by generating a standard curve using a recombinant plasmid containing the CFAV or *rps6* segment insertion. Primers used for RT-qPCR detection are given in Additional file 1: Table S1.

### Mosquito propagation

**Fig. 1:**
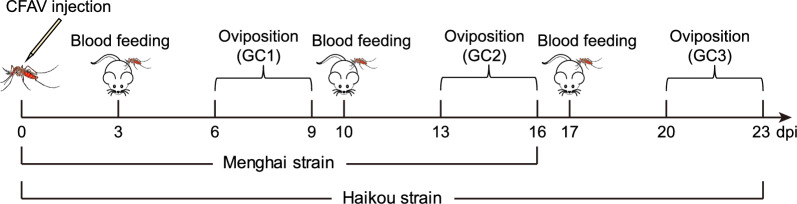
Schematic diagram of cell fusing agent virus (*CFAV*) injection and propagation of *Aedes aegypti* mosquitoes. *GC* Gonotrophic cycle,* dpi* days post-infection

Two hundred CFAV-infected parental female *Ae. aegypti* (F0) were allowed to blood-feed on Kunming mice (Beijing Vital River Laboratory Animal Technology, Beijing, China) for propagation on 3, 10 and 17 days post-infection (dpi). The mosquitoes were provided with filter paper placed in a small container with water for oviposition on 6–9 dpi [the first gonotrophic cycle (GC), GC1], 13–16 dpi (GC2) and 20–23 dpi (GC3) (Fig. 1). Eggs of Menghai strain from GC1 and GC2, and eggs of Haikou strain from all three GCs, were collected and hatched to produce progenies (F1), which were tested for CFAV infection on different days post-emergence (dpe).

### Statistical analysis

The data were visualized and analyzed using GraphPad Prism version 9.4 (GraphPad Software, San Diego, CA). The survival curves of *Ae. aegypti* after PBS or CFAV injection were compared by Kaplan–Meier survival analysis with a log-rank (Mantel-Cox) test. Normality and heteroscedasticity of residuals were evaluated by Shapiro–Wilk (*W*) test and Spearman’s test, respectively. The viral loads in the ovary and carcass were compared by two-tailed Mann–Whitney test. *P* < 0.05 was considered to indicate statistical significance.

## Results

### Sequencing and phylogenetic analysis of CFAV isolated from Aag2 cells

The CFAV used in this study was directly harvested from the Aag2 cells. The complete genome was amplified by using 16 pairs of primers and RACE, and the products were sequenced by Sanger sequencing. There were mutations at sites 3429 and 3444 of the open reading frame (ORF). These may have been T or C at ORF3429 and A or C at ORF3444, leading to four possible combinations (Additional file 2: Fig. S1). The segment containing these two sites was cloned into a linear vector and the recombinant plasmid was sequenced. The virus stock consisted of three strains, named strain BJ01, BJ02, and BJ03 in this study, the respective proportions of which were 51.4%, 28.6% and 20.0%. The two mutations were located at the third position of the corresponding codon and did not change the encoded amino acids as they were nonsense mutations (Table 1). The virus stock used to infect mosquitoes in this study was a mixture of these three strains.

**Table 1:** Information on the three cell fusing agent virus (CFAV) strains harvested from the Aag2 cells

Strain	ORF3429			ORF3444			Number of clones	Percentage	Accession no.
	nt	Codon	aa	nt	Codon	aa			
BJ01	C	AUC	I	A	CUA	L	18	51.4%	NMDC60064201
BJ02	T	AUU	I	C	CUC	L	10	28.6%	NMDC60064202
BJ03	T	AUU	I	A	CUA	L	7	20.0%	NMDC60064203

*ORF* Open reading frame, *nt* Nucleotide, *aa* amino acid, *I* isoleucine, *L* leucine

**Fig. 2:**
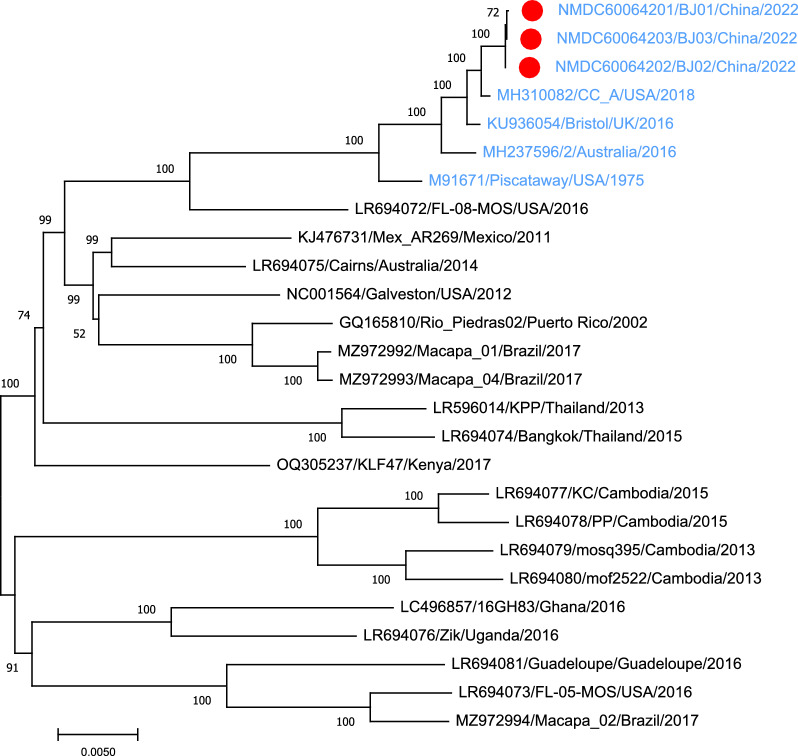
Neighbor-joining phylogenetic tree based on nucleotide sequences alignments of 26 CFAV sequences. Newly identified CFAV strains in this study are indicated by a* red dot*. CFAV strains* in blue* were derived from the Aag2 cell line and the others from *Aedes aegypti* mosquitoes. The* scale bar* indicates the evolutionary distance in number of substitutions per nucleotide, and the level of principal bootstrap support is indicated

In the phylogenetic analysis, the three CFAV strains isolated in this study clustered together with those from *Ae. aegypti* cell lines but far from those isolated or identified from *Ae. aegypti* mosquitoes (Fig. 2).

### Infection with exogenous CFAV did not influence the survival of *Ae. aegypti*

**Fig. 3:**
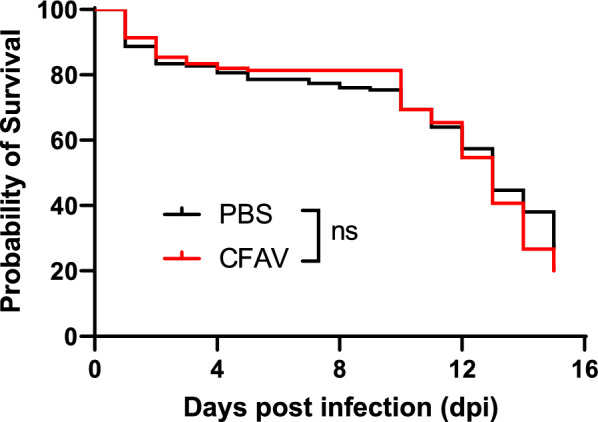
Survival curve of *Aedes aegypti* after intrathoracic (IT) injection. Female *Ae. aegypti* mosquitoes (Menghai strain) were intrathoracically injected with CFAV or phosphate buffered saline (PBS) (*n* = 150 in each group) and the survival of mosquitoes within 15 days was monitored. Survival curves of the two groups were compared by Kaplan–Meier survival analysis with a log-rank (Mantel-Cox) test. *ns* No significant difference

To examine whether the exogenous CFAV strains isolated from the cells would be deleterious to the mosquito, 150 female *Ae. aegypti* Menghai strain were inoculated with CFAV via IT injection and the subsequent number of dead mosquitoes was recorded daily. Mosquitoes injected with PBS were used as controls. The median survival of the two groups was 13 days and there was no significant difference between them [Fig. 3; log-rank (Mantel-Cox) test, *χ*^2^ = 1.217, *df* = 1, *P* = 0.2699], indicating that CFAV isolated in the Aag2 cells was not deleterious to the *Ae. aegypti* mosquitoes.

### CFAV replication in parental *Ae. aegypti* after injection

**Fig. 4:**
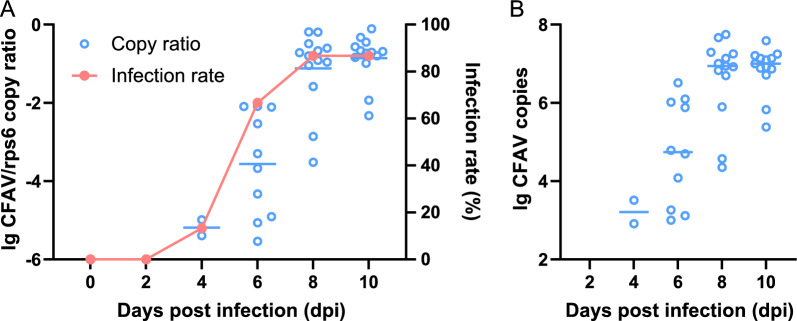
Replication kinetics of CFAV in the parental *Aedes aegypti* mosquitoes after IT injection. Four hundred female *Ae. aegypti* mosquitoes (Menghai strain) were intrathoracically injected with CFAV stock and the viral RNA and* rps6* expression on the indicated days were examined by reverse transcription-quantitative polymerase chain reaction (RT-qPCR) (*n* = 15 on each day). **A** The CFAV load was normalized to the host* rps6* messenger RNA and the results are expressed on a log10 scale.* Blue circles* represent the CFAV load and the mean values are indicated by the* horizontal line* for each group. The CFAV infection rate (IR) is presented as a* red line*. **B** The CFAV copies per mosquito are shown as* blue circles*. For other abbreviations, see Figs. 1 and 3

After IT injection of CFAV, viral RNA was detected in the whole body of *Ae. aegypti* Menghai strain on 0, 2, 4, 6, 8 and 10 dpi. Viral RNA was undetectable on 0 and 2 dpi. However, the virus replicated explosively, 10,000 times from 4 to 8 dpi, with the mean load (the common logarithm of the CFAV/*rps6* copy ratio) increasing from -5.19 to -1.12 and finally reaching -0.86 on 10 dpi (Fig. 4A). The infection rate (IR) also rapidly increased, from 13.3% on 4 dpi to 86.7% on 8 and 10 dpi (Fig. 4A). However, it should be noted that the low IR in the early stage of infection was due to the low sensitivity of the RT-qPCR examination and did not indicate that most of the mosquitoes in the group were not infected with CFAV. The total number of CFAV copies in each mosquito reached a median of 10^7^ copies/mosquito on 10 dpi (Fig. 4B).

### CFAV did not vertically transmit to the filial *Ae. aegypti* from infected parental *Ae. aegypti*

The progeny (F1) in two of the GCs of CFAV-infected parental *Ae. aegypti* Menghai strain (F0) were examined for CFAV infection on different days post-emergence. Twenty females and 20 males from both GC1 and GC2 were tested by RT-qPCR on 3 dpe, and 20 females from GC1 and GC2 were tested by RT-qPCR on 10 dpe, but none of them were virus positive. To examine whether blood-feeding would promote virus replication, the mosquitoes were fed with blood on 10 dpe, and 20 females from GC1 and GC2 were examined, respectively, 3 days later. However, the IR remained at zero (Table 2).

**Table 2:** Infection rate of CFAV in the progeny from different gonotrophic cycles (*GC*) of *Aedes aegypti* Menghai and Haikou strains

Strain	dpe	Gender	GC		
			1	2	3
Menghai	3	F	0/20	0/20	–
		M	0/20	0/20	–
	10	F	0/20	0/20	–
	13	F	0/20	0/20	–
Haikou	5	F	0/20	0/20	0/20
		M	0/20	0/20	0/20

*dpe* Days post-emergence, *F* female, *M* male

The endogenous viral elements (EVE) derived from CFAV (CFAV-EVE) in the genome of *Ae. aegypti* may limit CFAV replication in the ovary through the PIWI-interacting RNA pathway [16]. The Menghai strain carried CFAV-EVE at a rate of 100% in the population (Additional file 3: Fig. S2), which may have inhibited CFAV from invading the ovary and being transmitted to the offspring. The experiment was repeated using *Ae. aegypti* Haikou strain, which does not carry CFAV-EVE in its genome (Additional file 3: Fig. S2). The Haikou strain was inoculated with a much higher load of CFAV, which had been amplified in C6/36 cells, and was allowed to oviposit for a total of three GCs. Twenty females and 20 males from three GCs were tested for CFAV infection on 5 dpe and all were found to be CFAV free (Table 2).

### CFAV copies were much lower in the ovarian tissues than in other tissues

**Fig. 5:**
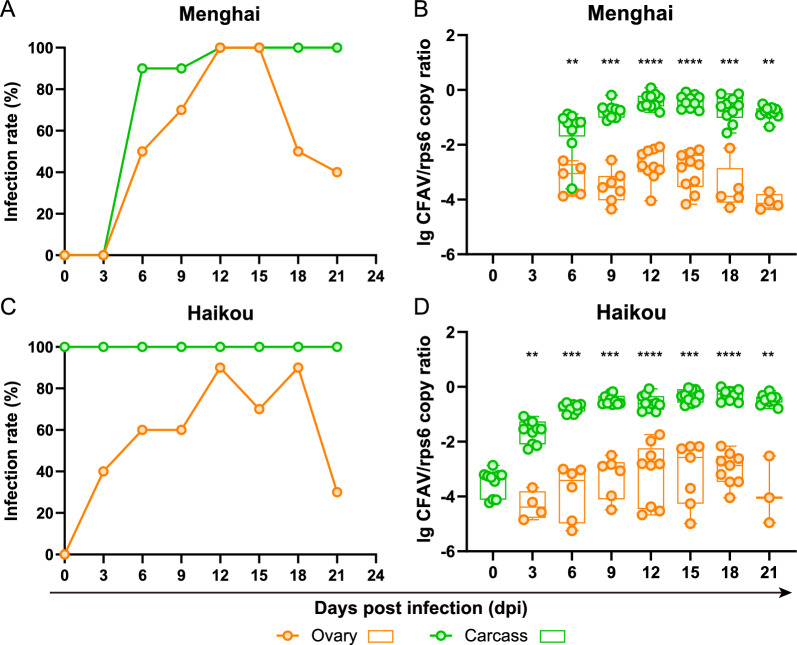
Replication kinetics of CFAV in different tissues of *Aedes aegypti* after IT injection. *Aedes aegypti* was dissected on the indicated days post-infection, and the ovary and carcass were examined for CFAV infection by RT-qPCR (*n* = 10 on each day). IR of CFAV in the ovary and carcass of *Ae. aegypti* Menghai strain (**A**) or Haikou strain (**C**). CFAV copies normalized to host* rps6* mRNA in the ovary and carcass of *Ae. aegypti* Menghai strain (**B**) or Haikou strain (**D**). Each* filled circle* represents an individual sample, and only viral RNA positive values are shown. Box plots show the median and the 25-75th percentiles, and the whiskers denote the maximum and minimum values. As the residuals of the viral loads did not pass normality and heteroscedasticity tests, they were analyzed by two-tailed Mann–Whitney test. ** *P* < 0.01, *** P < 0.001, **** *P* < 0.0001

To further investigate why vertical transmission of CFAV to the progeny failed, new batches of *Ae. aegypti* Menghai and Haikou strains were inoculated with the virus stock. CFAV copies were detected in the ovary and ovariectomized body (carcass) within 21 days of the injection. The IR of CFAV in the ovary and carcass of the Menghai strain remained at 0% for the first 3 days, similar to the previous result (Fig. 4). IR in the carcass rapidly increased to 90% on 6 dpi and then remained at a high level, 90–100%, afterwards. However, IR in the ovary increased only moderately, peaked at 100% on 12 ~ 15 dpi, and subsequently decreased to 40% by the endpoint of experiment on 21 dpi (Fig. 5A). In the Haikou strain, which was inoculated with a much higher virus dose, the IR in the carcass remained at 100% throughout the experiment, while the IR in the ovary increased moderately, peaked at 90% on 12 and 18 dpi, and decreased to 30% on 21 dpi, similar to the results for the Menghai strain (Fig. 5C). In the Menghai strain, the median CFAV/*rps6* copy ratio ranged from − 4.36 to − 2.08 in the ovary, and from -3.61 to -0.08 in the carcass. The viral load in the ovarian tissues was significantly lower than in other tissues (*U* = 0 or 3, *P* < 0.01) at 6–21 dpi (Fig. 5B). In the Haikou strain, the median CFAV/*rps6* copy ratio ranged from -5.25 to -1.74 in the ovary, and from -4.23 to 0.00 in the carcass. The viral load was also significantly lower in the ovarian tissues than in other tissues (*U* = 0, *P* < 0.01) at 3–21 dpi (Fig. 5D). This suggested that certain mechanisms might have limited CFAV replication in the ovary, leading to the failure of CFAV colonization in *Ae. aegypti*.

## Discussion

Since its discovery in an *Ae. aegypti* cell line almost 50 years ago, CFAV had been isolated or detected in *Ae. aegypti* mosquito populations from the Americas, Africa, Asia, and Oceania [17]. As an increasing number of studies have reported interactions between CFAV and other arboviruses, and it was shown that CFAV could reduce the competence of mosquitoes for specific arboviruses, CFAV may serve as a biocontrol agent to restrict the transmission of arboviruses [5, 6], e.g., as reported for *Wolbachia* [18, 19]. CFAV is much easier to cultivate and genetically engineer than *Wolbachia*, which makes it a much more promising biocontrol agent than the latter. We attempted to introduce Aag2-derived CFAV into *Ae. aegypti* mosquitoes by IT injection in the present study, to observe the effect of exogenous CFAV on the survival of *Ae. aegypti* and whether this virus can stably colonize populations of this mosquito.

CFAV inoculation did not affect the survival of *Ae. aegypti* (Fig. 3), and the virus could replicate normally, reaching a high IR and a stable viral load in the mosquito body (Fig. 4). However, the IR of CFAV in the offspring of the three GCs was 0% regardless of gender or engorgement (Table 2). Using the same method as employed here, Contreras-Gutierrez et al*.* [12] successfully generated an *Ae. aegypti* model stably carrying an exogenous CFAV strain. In their experiment, the comprehensive IR of CFAV was 30.0% in the F1 generation, and reached 74.3% in the F2 generation. This notable difference between the results of Contreras-Gutierrez et al*.*’s [12] and our study warrants further investigation.

To explore whether CFAV had invaded the ovary, we injected another batch of mosquitoes and examined the number of viral copies in the ovary and ovariectomized body. In the ovary, the virus first multiplied and then was cleared, and the viral load was significantly lower than in the other tissues (Fig. 5). The mosquito ovary mainly consists of oocytes, nurse cells and follicular epithelium [20, 21], and the successful infection of oocytes is a prerequisite for vertical transmission to offspring. To determine the distribution of CFAV within the ovary more accurately and whether it successfully infect the oocytes, the use of immunofluorescence detection is recommended for future studies. If it is demonstrated that CFAV cannot invade the oocytes from the hemolymph, the injection of male *Ae. aegypti* with CFAV might serve as an alternative method, as it has been shown that CFAV can be passaged by paternal vertical transmission at a filial IR of 85% [22].

The genetic backgrounds of mosquitoes greatly influence their susceptibility to viruses [10]. For example, EVE cognate to CFAV in the genome of *Ae. aegypti* could limit CFAV replication in the ovary [16]. However, in the present study, the results were similar for the CFAV-EVE-carrying Menghai strain and the CFAV-EVE-free Haikou strain: the viral load was significantly lower in the ovary and the virus could not be vertically transmitted in either strain. Whether the *Ae. aegypti* Bangkok strain used by Contreras-Gutierrez et al*.* [12] carried CFAV-EVE in its genome was unclear, but it seemed that CFAV-EVE was not the key factor that prevented CFAV transmission. Zakrzewski et al*.* [23] detected CFAV through RNA sequencing in *Ae. aegypti* captured in Bangkok, Thailand, in 2015, and the CFAV-free *Ae. aegypti* used by Contreras-Gutierrez et al. [12] was also obtained from Bangkok, in 2011, suggesting that *Ae. aegypti* in that area may have been somewhat susceptible to CFAV. To the best of our knowledge, CFAV has not been detected in or isolated from wild caught *Ae. aegypti* in China, which may indicate that the two *Ae. aegypti* strains used in this study are more resistant to this virus. However, resistant genes specific to this virus have yet to be discovered.

Different strains of a virus may also significantly influence the results of a study. The CFAV strain (NC001564) used by Contreras-Gutierrez et al. [12] was isolated from the *Ae. aegypti* Galveston strain, whereas the CFAV used in this study was isolated from Aag2 cells, which were previously reported to be persistently infected with this virus [7, 24]. The phylogenetic analysis revealed a significant genetic difference between these two strains (Fig. 2), indicating that their evolution has diverged since the establishment of Aag2 cell line from *Ae. aegypti* embryos in 1968 [25]. The trade-off hypothesis proposes that the fitness of a virus increase in one host usually diminishes in the other [26]. This hypothesis was widely supported by the effects of the serial passaging of a virus in one cell line for the production of vaccines that are attenuated to humans [27–29]. Thus, the fitness of CFAV might have been lower in *Ae. aegypti* due to decades of passaging in the Aag2 cell line.

An important genomic difference between Aag2-derived and *Ae. aegypti*-derived CFAV is that the latter encodes an additional fairly interesting *Flavivirus* ORF (FIFO) protein through a -1 programmed ribosomal frameshift (PRF), whereas the FIFO ORF in the former is interrupted by premature stop codons [30]. PRF is utilized by many viruses to increase the coding capacity within a limited viral genome, and the related translation product always benefits virus infection of the host. For example, NS1’, a PRF product of Japanese encephalitis virus, enhanced the viral infection of dendritic cells and macrophages [31], while the live-attenuated vaccine SA_14_-14–2 strain, which had lost NS1’ expression, showed slower replication kinetics in cells and was essentially non-neuroinvasive and non-neurovirulent in weanling ICR mice [32]. A transcription factor protein produced by alphaviruses via a -1 PRF played a role in viral assembly, as transcription factor-deficient virus exhibited reduced virion release [33] and attenuated pathogenesis [34–36]. A similar phenomenon was observed in other viruses, such as human immunodeficiency virus [37], influenza A viruses [38] and coronaviruses [39, 40]. In future studies, we intend to explore whether, and if so how, the FIFO protein benefits the colonization of *Ae. aegypti* mosquitoes by CFAV, and the effects of this protein on other arboviruses that co-infect mosquitoes.

Elucidating what prevents CFAV from colonizing *Ae. aegypti* by artificial infection could aid the establishment of *Ae. aegypti* colonies that carry exogenous CFAV and also provide a deeper understanding of antiviral mechanisms in mosquitoes, both of which could be useful for the development of mosquito-borne disease prevention and control programs.

## Conclusions

We isolated three new strains of CFAV from Aag2 cells but were unsuccessful in our attempt to introduce them into *Ae. aegypti* mosquitoes to establish a model that stably carries exogenous CFAV. Aag2-derived CFAV replicated efficiently in the carcass of *Ae. aegypti*, but the viral load was much lower in the ovary and the virus failed to infect the filial generation. We speculate that the Aag2-derived CFAV had lost the ability to invade the ovary due to FIFO protein deficiency and thus could not be vertically transmitted in *Ae. aegypti*. These results provide new insights for future studies on how CFAV and other ISVs colonize and are vertically transmitted in mosquitoes.

### Supplementary Information

**Additional file 1: Table S1.** Primers used in this study.

**Additional file 2: Figure S1.** Two mutation sites in the 6th polymerase chain reaction (PCR) segment.

**Additional file 3: Figure S2. **PCR detection of cell fusing agent virus-endogenous viral element in *Aedes aegypti* Menghai and Haikou strains.

## Data Availability

All data generated or analyzed during this study are included in this published article and its additional files. The genomic sequences of the three CFAV strains have been deposited in the China National Microbiology Data Center (NMDC) under accession numbers NMDC60064201-NMDC60064203.

## Abbreviations

CFAV: Cell fusing agent virus
DENV: Dengue virus
dpe: Days post-emergence
dpi: Days post-infection
EVE: Endogenous viral elements
FIFO: Fairly interesting *Flavivirus* open reading frame
GC: Gonotrophic cycle
IR: Infection rate
ISV: Insect-specific virus
IT: Intrathoracic
ORF: Open reading frame
PCLV: Phasi Charoen-like virus
PCR: Polymerase chain reaction
PRF: Programmed ribosomal frameshift
RACE: Rapid amplification of complementary DNA ends
RT-qPCR: Reverse transcription-quantitative polymerase chain reaction
ZIKV: Zika virus
