# Supplementary figures and images for: Cell fusing agent virus isolated from Aag2 cells does not vertically transmit in Aedes aegypti via artificial infection
Source: Parasit Vectors. 2023 Nov 6;16:402. doi: 10.1186/s13071-023-06033-3 (PMC10626676; doi:10.1186/s13071-023-06033-3)

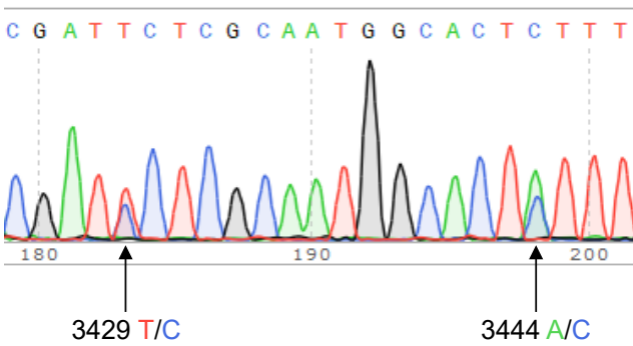

Supplement: Supplementary file 2 — Additional file 2: Figure S1. Two mutation sites in the 6th polymerase chain reaction (PCR) segment. [file 13071_2023_6033_MOESM2_ESM.pdf]

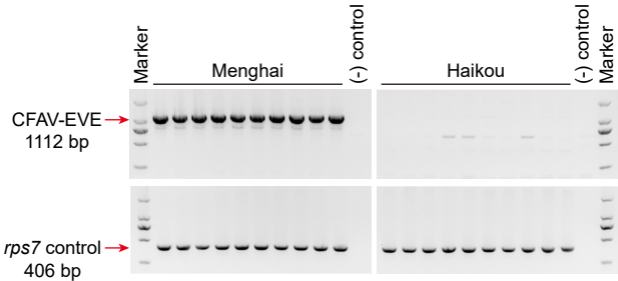

Supplement: Supplementary file 3 — Additional file 3: Figure S2. PCR detection of cell fusing agent virus-endogenous viral element in Aedes aegypti Menghai and Haikou strains. [file 13071_2023_6033_MOESM3_ESM.pdf]
